# Operationalising the Global Financing Facility (GFF) model: the devil is in the detail

**DOI:** 10.1136/bmjgh-2018-001369

**Published:** 2019-03-22

**Authors:** Nicole A Salisbury, Gilbert Asiimwe, Peter Waiswa, Ashley Latimer

**Affiliations:** 1 PATH, Seattle, Washington, USA; 2 Infectious Diseases Research Collaboration, Kampala, Uganda; 3 School of Public Health, Makerere University, Kampala, Uganda

**Keywords:** health policy, health systems, health systems evaluation

**Summary box**

- Recent modelling exercises project that if fully funded, the Global Financing Facility could contribute to significant health gains for women and children, and by doing so help to facilitate the achievement of the health-related Sustainable Development Goals.
- Operationalisation of this approach is sure to be complicated by myriad global-level and country-level variables that affect implementation, and there are many lessons to be learnt from the experiences of Gavi and the Global Fund as they pertain to design, implementation, monitoring, evaluation and learning.
- Careful coordination across funding mechanisms, at all levels (from global to country), will be crucial to achieving impact, and should be institutionalised into donor processes.
- The Global Financing Facility should embed opportunities for evaluation and learning at all stages to contribute to process improvement as the model evolves.

## Introduction

In 2015, at the Third International Financing for Development Conference, world leaders launched the Global Financing Facility (GFF). The goal of the GFF is to ‘accelerate efforts to end preventable maternal, newborn, child and adolescent deaths and improve the health and quality of life of women, adolescents and children’.^1^ The GFF is a country-led financing approach that seeks to align funding sources, including public and private, domestic and external, for priority interventions to address the health of women and children. Unlike other multilaterals, the GFF provides only modest amounts of grant funding to leverage other development assistance funding (including loans) and domestic resources to prioritise a country’s most pressing reproductive, maternal, newborn, child and adolescent health and nutrition (RMNCAH-N) needs.

An article recently published in this journal^2^ highlights the enormous potential of the GFF model, if fully funded, to close the financing gap and contribute to significant health gains. Chou *et al* 2 estimate that with $2.6 billion of GFF Trust Fund resources, the GFF partnership could contribute to mobilising $50–75 billion in additional funds to scale up priority interventions. Dependent on available resources, between 11.9 and 12.4 million maternal, neonatal and child deaths, and 97.7–104.1 million cases of stunting, could be averted, relative to a continuation of historical trends.

These projected results are encouraging. However, experience from other global financing mechanisms indicates that operationalisation at the country level is complicated, particularly as funders mature and evolve. Achieving the impact modelled by Chou *et al* 2 depends not just on the allocation of sufficient resources, but equally critically on a comprehensive, country-led investment case to identify and prioritise evidence-based interventions to improve outcomes, and on many implementation-related variables, including crucial adaptations and course corrections throughout. This will require careful work at all stages of the investment life cycle. Civil society organisations (CSOs) have an especially important support role to play in navigating each of these areas.

The GFF can learn from existing funders (and indeed to generate further learnings). To this end, this commentary draws findings from prospective, multiyear evaluations of Gavi, the Vaccine Alliance (the Gavi Full Country Evaluations),^3^ and The Global Fund to Fight AIDS, Tuberculosis and Malaria (the Global Fund Prospective Country Evaluations).^4^ Thoughtful coordination across these entities, as well as together with the World Bank, will be essential to reduce inefficiencies, ensure harmonisation and catalyse greater impact. The GFF Investors Group, and Gavi and Global Fund Boards, which all of the above funders are party to, provide platforms for such global-level coordination.

## Global policies and communication

Gavi and the Global Fund are learning organisations engaged in a constant cycle of policy design, implementation, evaluation and redesign. The drivers of these reforms vary but are often attributed to a desire to foster greater country ownership and to simplify donor processes, thereby reducing transaction costs and increasing value for money. A consequence of this constant change is confusion at the country level in relation to policies and financing requirements, which undermines (at least short-term) efforts at simplification.

Gavi and the Global Fund have different (and evolving) models to help with communication and policy translation. Similar to GFF, Gavi started off as a ‘lean’ Secretariat, reliant primarily on alliance partners (eg, WHO, Unicef, World Bank). As the complexity associated with Gavi grew, so too did the Secretariat. Gavi now relies not just on partners, but increasingly on a growing number of ‘senior country managers’ (SCMs) who interface with country-level counterparts. The effectiveness of this approach varies from country to country but has generally improved over time as SCMs are hired with technical and policy, in addition to grants management, backgrounds. The Global Fund uses Geneva-based country teams, which comprised fund portfolio managers, programme officers, and monitoring and compliance staff, that play a prominent role in grants management throughout the grant life cycle. This is perceived as strengthening the quality of funding requests, improving the efficiency of grantmaking processes, and facilitating the implementation of strategies and policies at the country level. The GFF, which has introduced liaison officers to support proactive engagement with all stakeholders at the country level, complementing World Bank country teams, can as a country-led model and platform help align and prioritise collective efforts.

As a relative newcomer, a lack of stakeholder awareness of the GFF processes is unsurprising and will require a concerted policy translation effort. As it continues to mature, a strategy for ensuring relevant stakeholders, especially ministries of health and finance donors, and local decision makers, are up to speed with each iteration is essential. The GFF Secretariat is small and aims to remain lean, and therefore has limited capacity to conduct the level of engagement required for a new, evolving funding mechanism. Partners, including CSOs, are pivotal to bridging this gap, both at the global and country levels.

## Programme design

Achieving impact depends on a robust investment case (based on a compelling theory of change) that correctly identifies the most pressing RMNCAH-N bottlenecks, and the mix of interventions to address them. Indeed, available investment case guidance states that ‘long-term transformational’ reforms (eg, introduction of a basic benefits package) should be prioritised alongside singular interventions.^5^

The design of Gavi Health Systems Strengthening (HSS) investments often require technical assistance (TA). Given the many competing priorities for short-staffed teams and the perceived complexity of the application process, the availability of TA was largely viewed as essential; however, concerns exist about sustainability, especially in the absence of an explicit focus on strengthening local capacities.^6^ Similarly, Global Fund requests involve extensive support; for example, in the Democratic Republic of the Congo, more than 20 consultants supported the recent tuberculosis/HIV application.^7^

Too often there are missed opportunities for harnessing synergies across the investments of different funders. Stakeholders in Uganda devoted significant resources, during a 6-month period in 2015–2016, to developing a Gavi HSS proposal.^8^ Despite considerable potential for overlap, the Global Fund application process, which included investments in Resilient and Sustainable Systems for Health funding, occurred the following year, in 2017.^9^ Better harmonisation of timing and content for funding proposals is critical.

## Implementation

Successful implementation partially depends on funds that are timely and predictable, with funding cycles well aligned to country systems. In the cases of Gavi and the Global Fund, timely implementation has been undermined by time-consuming and bureaucratic processes. Both donors emphasise mitigating risk of financial mismanagement, but the requirements associated with securing funds are perceived by country stakeholders as burdensome.

For instance, implementation of Global Fund grants is often delayed by protracted selection processes for subrecipient implementers and establishing agreements with other ministries that support implementation. Access to Gavi HSS funds sometimes takes years. Mozambique received their first disbursement in 2016 for a grant approved in 2012. Although the delay meant the design of the grant was outdated, stakeholders were reluctant to reprogramme because of the associated transaction costs.^10^ This delays health impact. Because the GFF investment cases are intended to mobilise resources from multiple donors and domestic sources, ensuring the timely availability of funds may prove particularly challenging given different donor requirements. As all GFF Trust Fund grants are channelled through World Bank operations through the government, the GFF can support aligning and ensuring timely processing and implementation of funds.

## Monitoring, evaluation and learning

Demonstrating results and measuring impact will be especially important to ensure ongoing investments through the GFF Trust Fund. This is a key area for Gavi and the Global Fund also as their donors have become increasingly keen to track investment impact. Achieving a reasonable balance between reporting requirements and the level of required effort is essential. In an effort at simplification, Gavi replaced separate monitoring and reporting requirements with single grant performance frameworks which define key metrics for monitoring the performance of grants. GFF uses core indicators aligned with the Sustainable Development Goals and based on country priorities and available data, but there remains opportunity for harmonisation of requirements across all donors to reduce reporting burdens.

Furthermore, improving implementation and impact requires an understanding of complex results chains for each investment with a focus on where the process could be strengthened. The development and early implementation of investment cases in a multitude of countries present an important opportunity to document learnings, with an emphasis on process improvement, especially for countries yet to join the GFF, and the GFF should look for further opportunities to embed learning in its processes.

## Conclusion

Established financing mechanisms, such as Gavi and the Global Fund, have generated many lessons that can help the GFF to replicate best practices and avoid common pitfalls. Indeed, as the GFF itself matures, it will generate further findings and insights. Early observations suggest that coordination is dependent on specific individuals who operate across the three entities, and that these linkages could be more formally institutionalised, even beyond the GFF Investors Group and board membership. Thoughtful coordination, across funding mechanisms at all levels, from country implementers to global leaders, must be built into donor processes.

As illustrated by Chou *et al*, the GFF is designed to catalyse significant improvements in the health of women and children. The real work that achieves impact requires not just financial, but also technical inputs, from diverse stakeholders, including ministries of health and finance, donors, the private sector, technical partners and CSOs. Global financing entities such as Gavi, the Global Fund, the GFF and other financiers must work together, and learn from each other, as they work towards their distinct, but complementary goals.
